# Pine Rhizosphere Soil Microorganisms Enhance the Growth and Resistance of *Pinus massoniana* Against Nematode Infection

**DOI:** 10.3390/microorganisms13040790

**Published:** 2025-03-30

**Authors:** Jiacheng Zhu, Chenxi Deng, Yichi Zhang, Manman Liu, Guoying Zhou, Junang Liu

**Affiliations:** 1 Key Laboratory of National Forestry and Grassland Administration on Control of Artificial Forest Diseases and Pests in South China, Central South University of Forestry and Technology, Changsha 410004, China; 20200100002@csuft.edu.cn; 2 Hunan Provincial Key Laboratory for Control of Forest Diseases and Pests, Central South University of Forestry and Technology, Changsha 410004, China; 20221100108@csuft.edu.cn; 3 Key Laboratory of Cultivation and Protection for Non-Wood Forest Trees, Central South University of Forestry and Technology, Changsha 410004, China; 20231200064@csuft.edu.cn (Y.Z.); 20211100011@csuft.edu.cn (M.L.)

**Keywords:** pine wilt disease, soil microorganisms, growth-promoting, defense response mechanisms

## Abstract

Pine wilt disease, caused by *Bursaphelenchus xylophilus*, poses severe ecological and economic threats to coniferous forests. This study isolated two fungal (*Arthropsis hispanica*, *Penicillium sclerotiorum*) and two bacterial (*Bacillus amyloliquefaciens*, *Enterobacter hormaechei*) strains from *Pinus massoniana* rhizospheres, evaluating their biocontrol potential against pine wood nematodes. Molecular characterization confirmed strain identities. In vitro assays demonstrated that combined fermentation filtrates of CSX134+CSZ71 and CSX60+CSZ71 significantly enhanced plant growth parameters (height, biomass) and root-associated soil enzyme activities (urease, acid phosphatase) in *P. massoniana*. Treated plants exhibited elevated defense enzyme activities and upregulated defense-related gene expression. The treatments achieved 75.07% and 69.65% nematode control efficacy, respectively, compared to controls. These findings highlight the potential of microbial consortia in activating systemic resistance and suppressing pine wilt disease through the dual mechanisms of growth promotion and defense induction.

## 1. Introduction

Pine wilt disease poses a significant threat to pine trees and has spread extensively worldwide to areas including Japan, China, Korea, and Europe [1,2,3,4,5]. The occurrence of this devastating disease arises from intricate interactions among the *Bursaphelenchus xylophilus*, nematode vector of the *Monochamus* genus, and various host species belonging to the *Pinus* genus [6]. Globally, about 62 tree species are susceptible to the pine wood nematode, encompassing prominent host species in China such as *Pinus thunbergii* Parl., *P. massoniana* Lamb., and *Pinus densiflora* Sieb., among others [7,8,9,10].

*P. massoniana* holds critical significance in China as a valuable source of timber and resin. However, it stands as the main host species for pine wilt disease [11]. Current strategies for managing pine wilt disease primarily entail physical, chemical, and biological control methods. Physical control measures involve the interception of the vector nematode and the felling of infected trees, as well as the thermal or incineration treatment of infested timber [12]. Chemical control approaches commonly employ nematicides such as fosthiazate, oxamyl, abamectin, and imidacloprid [13]. Nonetheless, recent research has increasingly underscored the ecological disruptions caused by chemical control methods and the associated risks of pesticide residues to human health and wildlife [14]. Consequently, there has been a burgeoning interest in exploring biological control as an environmentally sustainable alternative.

Biological control involves the utilization of natural biological agents, such as bacteria and fungi, to contain plant diseases. Rhizosphere microorganisms are a group of beneficial root-colonizing microorganisms that inhibit plant pathogens through toxin production, biosurfactant secretion, and enzyme degradation, while also inducing host plant resistance and promoting plant growth [15]. The *Bacillus cereus* YN917 isolated by Zhou not only showed significant antifungal activity against *Magnaporthe oryzae*, but also has the ability to secrete a variety of plant growth-promoting and metabolic substances, which can promote the growth and resistance of hosts to diseases under greenhouse conditions [16]. Studies have shown that fungi and bacteria exhibit biocontrol effects against pine wood nematodes. Paiva et al. isolated strain 020 and RBT-200701 from *Bacillus subtilis*, which demonstrated nematicidal activity against the nematode [17]. Furthermore, three *Bacillus spp.* strains with strong antagonistic activity against the nematode were screened from the stem of pine trees [18]. Research by Berendsen et al. suggested that a combination of multiple antagonistic microbial strains showed better growth-promoting effects on plants compared to single strains [19]. These microbial consortia are typically composed of various rhizosphere microorganisms that interact and synergistically exert antagonistic effects on pathogens while promoting plant growth. For instance, the combined application of *Trichoderma harzianum* and *Pseudomonas fluorescens* effectively controlled pepper phytophthora blight [20], and the co-application of multiple *Trichoderma* spp. strains showed synergistic effects against onion root rot [21]. Additionally, the combined application of *P. fluorescens*, *B. subtilis*, and *Trichoderma viride* demonstrated good control efficacy against *Lasiodiplodia theobromae* [22].

In the field of biological control, some microbial strains have been identified as producers of metabolites that exhibit antagonistic effects against pathogens, thereby aiding in plant disease management. For instance, the metabolite plantazolicin derived from *Bacillus amyloliquefaciens* strain FZB42 demonstrates nematocidal activity [23]. Similarly, the application of the culture solution of *Enterobacter cloacae*, an endophytic bacterium that possesses nitrogen-fixing capabilities isolated from rice, significantly enhances rice resistance against sheath blight. This treatment also leads to alterations in enzyme activities associated with disease resistance, including LOX, POD, and PAL [24]. Biological control is a promising approach to control pine wood nematode disease. The objectives of this study were as follows: (1) to isolate two strains of fungi and two strains of bacteria exhibiting nematicidal activity; (2) to assess the growth-promoting properties of the fungi composite and their potential for the biocontrol of *P. massoniana*; and (3) to infer potential mechanisms through enzyme activity and the expression levels of defense-related genes in *P. massoniana*.

## 2. Materials and Methods

### 2.1. Sample Collection and Treatment

Soil materials for this study were collected in Tianxin District, Changsha City, Hunan Province (N28°07′05″, W112°59′47,731″; Gejia City, Liuyang City, N28°04′26″, W113°27′50″). Sampling occurred in October 2022. Soil samples were collected at a distance of 20 cm from the root system, with three sampling points at each distance and three biological replicates at each sampling point. The sampling tools were sterilized with alcohol to remove debris from the surface of the soil layer, and the collected soil was placed in sterile sampling bags and quickly stored on dry ice.

### 2.2. Microbial Strains

Fungal and bacterial strains previously isolated from rhizosphere soil were preserved in the laboratory.

### 2.3. Pine Wood Nematode

The PWN (pine wilt disease) pathogen *B. xylophilus* was isolated from diseased *P. massoniana* trees using the Baermann funnel method [25]. Pine wood nematodes were cultured by inoculating *B. xylophilus* in PDA solid medium and incubated in the dark at 25 °C for 7 days. Pine wood nematodes were inoculated onto gray *Staphylococcus* mycelium spread on plates and incubated in a dark at 25 °C for 5–7 days. The strain of *Botrytis cinerea* used in this experiment was provided by Zhejiang University of Agriculture and Forestry.

### 2.4. Medium

Bacterial medium: (1) Luria–Bertani (LB)—solid medium [26]; (2) Luria–Bertani (LB)—liquid medium [26]. Fungal medium: (1) PDA—solid medium [27]; (2) PDB medium [28].

### 2.5. Isolation and Screening of Pine Wood Nematode Fungi

For the isolation of fungi, 0.5 g of soil sample were placed in 45 mL sterile water. The suspension was incubated at 37 °C with agitation for 30 min. Subsequently, 1 mL of the soil suspension was used for soil dilution according to a previously reported method [27]. A total of 100 μL of each diluted soil sample was spread onto PDA solid medium. The plates were then inverted and incubated at 28 °C in a growth chamber. Daily observations were made to monitor fungal growth on the plates. Upon detection of new hyphae or colonies, they were immediately transferred to fresh plates for further purification until fungal isolates with distinct colony characteristics were obtained.

Screening for pine nematode-antagonistic fungi: the isolated and purified strains to be tested were inoculated into PDB liquid medium; then, the samples were placed in a constant-temperature incubation shaker rotating at 180 r/min and incubated at 28 °C for 5 d to obtain the fermentation product. The solution was centrifuged at 1500 rpm for 5 min. The nematode solution obtained by the Bellman funnel method was centrifuged at 1500 rpm for 5 min, and the supernatant was discarded after centrifugation; then, 1 mL of sterile water was added to keep the pine nematodes in suspension, and the procedure was repeated three times after centrifugation. The activity of the fermentation broth against pine wood nematodes was determined using the immersion method, as described [29]. The preparation method for the pine wood nematode suspension involved mixing the nematodes with sterile water to achieve a concentration of 2000 nematodes/mL. The suspension was utilized for screening nematode-antagonistic fungi and for conducting pot experiments.

The laboratory-cultivated sterile pine wood nematodes were prepared as a suspension at a concentration of 2000/mL via mixing with sterile water. The fermentation filtrate of the test strain and the nematode suspension were mixed in a 1:1 ratio. Each group consisted of three replicates, and the mixtures were placed in a 25 °C incubator. The mortality rate of the pine wood nematodes was observed at 24 h and 48 h. To verify the nematode mortality, a mixture of 3% NaCl and the nematode suspension was prepared in a ratio of 1:1 and allowed to stand for 10 min. Nematode mortality was determined by assessing the stiffness of the pine nematode’s body and its lack of response to physical stimulation [30]. The formula used to calculate the corrected nematode mortality rate (CM) is as follows: CM = (D − C)/(100 − C) × 100, where CM represents the corrected mortality rate (%), C represents the mortality rate of the control group (%), and D represents the mortality rate of the treatment group (%).

$$CM=\frac{D-C}{1-C}\times 100\%$$

### 2.6. Determination of the Nematicidal Activity of Fungal Fermentation Filtrates and Fungal Mycelia

The isolated and purified strains to be tested were inoculated into PDB liquid medium and placed in a 180 r/min constant-temperature incubation shaker; the fermentation solution was incubated at 28 °C for 5 d. The fermentation solution was obtained via centrifugation at 1500 rpm for 5 min. The nematode solution obtained by the Bellman funnel method was centrifuged at 1500 rpm for 5 min, and the supernatant was discarded after centrifugation. Then, 1 mL of sterile water was added to keep the pine nematodes in suspension and the procedure was repeated three times after centrifugation. The fermentation broth’s ability to kill pine wood nematodes was determined via the maceration method. The fungal mycelium was prepared as follows: take the mycelium after centrifugation, absorb 0.05 g into a sterile centrifuge tube with sterile paper, add 1 mL of sterile water, and shake at 5000 r/min for 5 min to make a mycelial suspension. The activity of the strain was determined using the maceration method described in Section 2.5.

### 2.7. Determination of Bacterial Fermentation Filtrates and Bacteriophage Nematicidal Activity

A total of 42 bacterial strains exhibiting nematicidal activity were isolated from the inter-root soil of *P. massoniana*, and inoculated in the LB liquid medium at constant temperature of 28 °C, in an oscillation box with 180 r/min culture for 2 d. The fermentation filtrate and suspension of 28 strains were used to determine the nematicidal activity of the strains.

Fermentation filtrate and bacterial suspension were prepared as follows: the strain was inoculated in 150 mL LB medium and incubated in a constant-temperature shaking box at 28 °C, 180 r/min, for 2 d. The supernatant was centrifuged at 10,000 r/min for 10 min at 4 °C with the help of centrifugation. The fermentation filtrate of the strain was obtained via filtration using a microporous filter membrane with a capacity of 0.22 µm, and was kept in storage for spare parts. The bacterial precipitate was resuspended in sterile water to make 10^7^ CFU/mL bacterial suspension. The method is the same as that shown in Section 2.5.

### 2.8. Molecular Biology Techniques for Identification Purposes

For the CSZ71 and CSZ33 fungal strains selected from the 2.6. screening, fungal colonies with a diameter of 5 mm were transferred to 150 mL conical flasks containing PDB medium and incubated at 28 °C with agitation for 5 days. Genomic DNA extraction was conducted using the Solaibao Fungal Genomic DNA Kit, following the manufacturer’s instructions. The fungal internal-transcribed spacer (ITS) region was then amplified using the universal primers ITS-1 and ITS-4 in a PCR reaction [31]. The PCR program consisted of an initial denaturation at 94 °C for 5 min, followed by 35 cycles of denaturation at 94 °C for 30 s, annealing at 56 °C for 30 s, extension at 72 °C for 1.5 min, and a final extension at 72 °C for 10 min. The amplified PCR products were subjected to agarose gel electrophoresis and sent to Shanghai Biotech Company for sequencing using the primers described earlier. The CSX134 and CSX60 bacterial strains selected from the screening were individually inoculated into LB liquid medium and incubated at 37 °C with agitation for 24 h. Genomic DNA extraction was performed using the Tian Gen Bacteria Genomic DNA Kit, following the manufacturer’s instructions. The bacterial total genomic DNA was then used as a template for PCR amplification using the universal primers 27F and 1492R, targeting the bacterial 16S rRNA gene [32]. The PCR reaction was carried out with an initial denaturation at 94 °C for 30 s, followed by 34 cycles of denaturation at 98 °C for 10 s, annealing at 55 °C for 30 s, extension at 72 °C for 1 min, and a final extension at 72 °C for 2 min. Following agarose gel electrophoresis of the amplified PCR products, the samples were sent to Shanghai Biotech Company for sequencing. The obtained sequencing data for both fungi and bacteria were subjected to BLAST analysis using the NCBI website (https://www.ncbi.nlm.nih.gov/, accessed on 25 February 2024). Finally, the MEGA11.0.13 software was employed to construct phylogenetic trees using the neighbor-joining method.

### 2.9. Determination of the Metabolites and Growth-Promoting Properties of Bacterial Strains

The measurement of chitinase, protease, amylase, and cellulase activities was conducted following the method described by Kakhki Et Al. [33]. CSX134 and CSX60 were inoculated into 100 mL sterilized LB liquid medium and incubated at 28 °C for 24 h. The fermentation broth was diluted with LB solution to a concentration of 10^8 CFU/mL. Subsequently, 2 µL of CSX134 and CSX60 fermentation broth was inoculated at three vertices of an isobilateral “∆” for protease, amylase, cellulase, and gibberellinase assays. CSZ71 and CSZ33 strains were cultured on PDA medium, and 5 mm fungal plugs were inoculated at the three vertices of an equilateral “∆” for protease, amylase, cellulase, and chitinase assays. Each experiment was conducted in triplicate, and plates were observed for the appearance of transparent zones within 2–4 days. Following the same protocol, 2 µL of CSX134 and CSX69 fermentation broth at a concentration of 10^8^ CFU/mL and 5 mm of CSZ71 and CSZ33 fungal plugs were inoculated onto Pikovskaya organophosphate-, Ashby-, and potassium-solubilizing assay plates. Plates were then incubated at 28 °C for 5 days to observe the formation of transparent zones. The indoleacetic acid (IAA) production ability was tested using Salkowski’s colorimetric method for the strain [34,35].

### 2.10. Screening of the Activity of Mixed Bacteria Against Pine Wood Nematodes

Strains 2.6 and 2.7. screened two strains of fungi and two strains of bacteria with pine wood nematode to determine their killing activity. Because their fermentation filtrate showed higher thread-killing activity than bacteria, the screened mixed bacteria were combined with the fermentation filtrate. The selected bacteria and fungi were combined in pairs, including “bacteria + bacteria”, “fungi + fungi”, and “bacteria + fungi” and a 1:1 fermentation filtrate combination was created. The method used to determine the thread-killing activity was the same as that presented in Section 2.5.

The two strains of CSX134 and CSX60 were inoculated in 150 mL LB medium and incubated at 180 r/min on a shaker at 28 °C for 48 h. The fermentation broth was centrifuged at 10,000 r/min for 10 min at 4 °C, and the supernatant was extracted and filtered through a microporous filtration membrane of 0.22 µm to obtain the fermentation filtrate of the strains, which was stored for future use. After the isolation and purification of CSZ71 and CSZ33 strains, they should be inoculated in PDB liquid medium and cultivated under constant-temperature oscillation box at 28 °C, 180 r/min, for 5 d. The culture solution was transferred to a 50 mL centrifuge tube and centrifuged under sterile conditions. The supernatant was subsequently centrifuged for clarification, and the fermentation filtrate of the fungal strains was then filtered using a 0.22 µm microporous membrane to obtain the assay line fermentation filtrate, which was stored for future use. A combined treatment was prepared by mixing 100 mL of fermentation filtrate, maintaining a 1:1 ratio.

### 2.11. Evaluation of the Growth-Promotion Using Mixed Bacteria on P. massoniana Seedlings

Selected *P. massoniana* seedlings were grown under controlled conditions with nutrient-rich soil, comprising a 3:1 mixture of field red loam soil. The soil was thoroughly mixed and uniformly moistened before sterilization at 121 °C for 30 min using an autoclave. Each pot was filled with 2000 g of the sterilized soil and placed in a greenhouse to acclimate the *P. massoniana* seedlings to stable growth prior to subsequent experiments. Detailed treatments are outlined in Table 1. The fermentation filtrates for each treatment group were prepared following the procedures described in Section 2.6 and Section 2.7. Seedlings were irrigated with 100 mL of sterile water mixed with 100 mL of the respective fermentation filtrate solution every 30 days. Control seedlings were irrigated solely with sterile water. Plant photographs and measurements were taken after 90 days of growth under these conditions.

### 2.12. Variation in Defense Enzyme Activities of P. massoniana Leaves After Different Treatments

Eighteen well-established *P. massoniana* seedlings were selected for subsequent experiments. Fermentation filtrates were prepared as described in Section 2.6 and Section 2.7, and sterile nematode suspensions were prepared following the method outlined in Section 2.5. The seedlings were allocated into different treatment groups, CSZ71, CSX134, CSXZ33, CSX60, CSZ71+CSX60, and CSZ71+CSX134, each consisting of three seedlings. Control groups included CK (three seedlings) and CK1 (three seedlings). Treatment groups received 100 mL of respective fermentation filtrates, while control groups were treated with 100 mL of sterile water. After 4 days, pine wood nematodes were inoculated using the patch method. Each treatment group and the fermentation filtrate groups were inoculated with 1 mL of nematode suspension (2000 nematodes/mL). Control group CK was inoculated with 1 mL of sterile water, and control group CK1 received 1 mL of sterile water mixed with 1 mL of nematode inoculum.

Pine needle samples were collected at 0, 12, 24, 48, 72, and 96 h post-treatment with fermentation filtrates from experimental groups. Additionally, samples were collected at 1, 3, 5, 7, 14, 21, and 28 days post-nematode inoculation in both treatment and control groups (CK and CK1). Peroxidase (POD), superoxide dismutase (SOD), and catalase (CAT) activities were assayed in inoculated leaves of both treatment and control groups using kits following the instructions from Suzhou Ge Ruisi Biotechnology Co., Ltd. (Suzhou, China)

### 2.13. Control of Pine Nematode Disease in P. massoniana Using Mixed Bacterial Agents

The treatment consisted of 30 biennial ponytail pines, while the control group had 10 biennial ponytail pines. Six treatment groups were established, CSZ71, CSZ33, CSX134, CSX60, CSZ71+CSX60, and CSZ71+CSX134, with the fermentation filtrate prepared as described in Section 2.6 and Section 2.7. Each treatment group received 100 mL of fermentation filtrate, while the CK control group received 100 mL of sterile water and the CK1 control group received 100 mL of sterile water. After 4 days, pine nematodes were inoculated using the skin-joining method: the fermentation filtrate group received 1 mL of inoculum containing 2000 nematodes, the CK control group received 1 mL of sterile water, and the CK1 control group received 1 mL of nematode inoculum.

### 2.14. RNA Extraction and Real Time-Quantitative Polymerase Chain Reaction (RT-qPCR)

The suspension of pine wood nematode was prepared and *P. massoniana* was inoculated following the skin-grafting method [36,37]. Two-year-old *P. massoniana* seedlings were used for inoculation against pine wood nematode. The fermentation filtrates of CSZ71, CSZ33, CSX134, CSX60, CSZ71+CSX60, and CSZ71+CSX134 were treated as the treatment groups, with three seedlings in each group. The control group CK had three seedlings and the control group CK1 also had three seedlings. For the fermentation filtrate treatment, 100 mL of fermentation filtrate was applied for single-strain treatment, while 100 mL of fermentation filtrate mixed at a 1:1 ratio was used for composite treatment. Control group CK was treated with 100 mL of sterile water, and control group CK1 received 1 mL of sterile water plus nematode inoculum. After a 4-day treatment period, inoculations of pine nematodes were performed using the skin-joining method. The fermentation filtrate treatment group was inoculated with 1 mL of nematode suspension containing 2000 nematodes, control group CK with 1 mL of sterile water, and control group CK1 with 1 mL of sterile water mixed with nematode inoculum. On days 1, 3, 5, 7, and 14 post-inoculation, pine needles from both treatment and control groups were frozen in liquid nitrogen for total RNA extraction. Each treatment and its corresponding control were replicated three times. The total RNA from *P. massoniana* needles was extracted using the RNAprep Pure polysaccharide polyphenol plant total RNA extraction Kit (Tiangen Biochemical Technology, Beijing, China), followed by reverse transcription with the AGRT Kit (cDNA first strand synthesis kit) to generate single-strand cDNA. Specific primers used included those for actin, PmCAT, PmSOD, and chitinase [38,39]; SYBR Green Pro Tag HS (Changsha Accurate Biotechnology Co., Ltd., Changsha, China) was utilized for PCR amplification on the QuantStudio 3 (Thermo Fisher Scientific (China) Co., LTD., Suzhou, China). PCR cycling conditions were identical to those specified by Yu et al. [40]. The gene expression levels were quantified using the 2^(-ΔΔCT) method. A quantitative real-time PCR analysis was conducted to assess relative gene expression levels, with data normalized against the Ct values corresponding to ACTIN. Primer synthesis was carried out by Shanghai Sangong Biological Co. (Shanghai, China)

### 2.15. Statistical Analysis

The data were processed using Microsoft Excel 2019 software and the processed data were analyzed for significance using IBM SPSS Statistics 26.0 software. Graphing was achieved using Graphpad prism 8 software. Duncan’s new multiple range test was used to detect significant differences between groups (*p*-value, *p* < 0.05), expressed as mean ± standard deviation.

## 3. Results

### 3.1. Isolation and Screening of Pine Wood Nematode-Killing Fungi and Bacteria

#### 3.1.1. Determination of Fungal Nematicidal Activity

**Figure 1:**
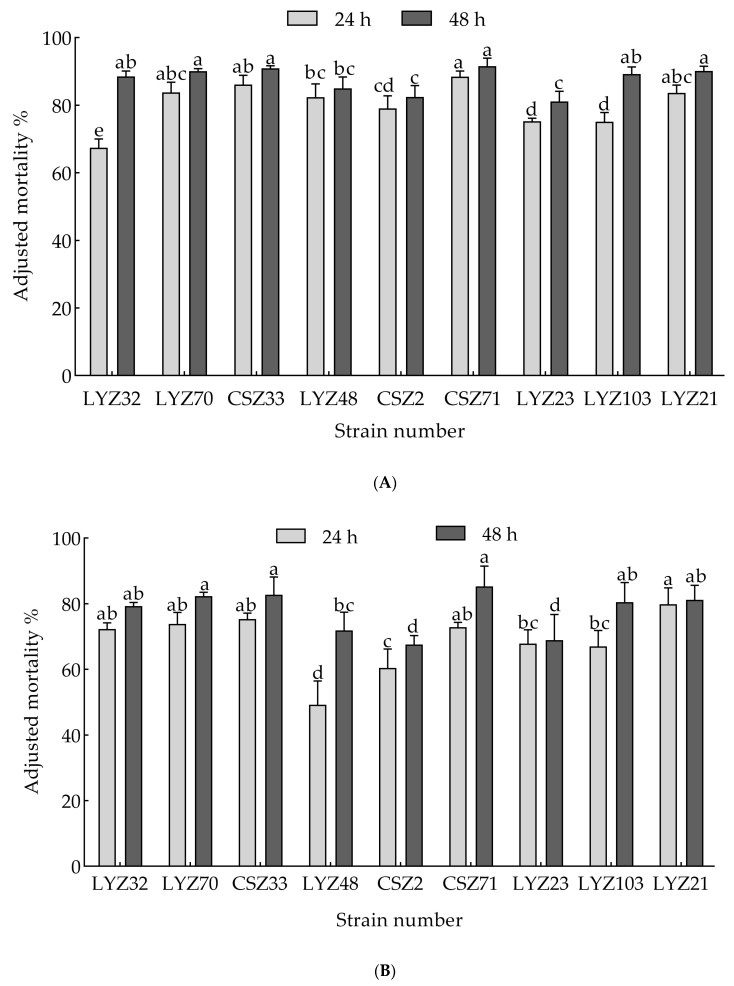
The nematicidal activity of fungal strains is presented, including (**A**) an assessment of 24-h and 48-h fungal fermentation filtrates, and (**B**) an evaluation of 24-h and 48-h fungal mycelial nematicidal activity. Significant variations in nematicidal efficacy among different strains were observed concurrently (*p* < 0.05).

A total of 106 strains of *P. massoniana* inter-root fungi were isolated through PDA solid medium, and the fermentation filtrate was used to determine the nematicidal activity of pine wood nematodes using the dip method of an initial screening and a re-screening. Following the determination of nematicidal activity, the fungal strains underwent assessments of the following parameters: (A) 24-h and 48-h fungal fermentation filtrates, and (B) 24-h and 48-h fungal mycelial nematicidal activity. Significant differences in nematicidal efficacy among strains were concurrently observed (*p* < 0.05). Subsequently, the nematicidal activities of fermentation filtrates and fungal mycelia from nine strains were evaluated, with the results depicted in Figure 1A,B. Overall, fungal fermentation filtrates exhibited higher nematicidal activity compared to fungal mycelia. Specifically, in Figure 1A, the fermentation filtrates from strains CSZ71 and CSZ33 showed the strongest 48-h nematicidal activity, achieving corrected mortality rates of 91.54% and 90.90%, respectively. Meanwhile, in Figure 1B, the 48-h nematicidal activity of the mycelia from strains CSZ71 and CSZ33 was the highest at 85.31% and 82.78%, respectively. Consequently, strains CSZ71 and CSZ33 were selected for further experimentation.

#### 3.1.2. Determination of Bacterial Fermentation Filtrate and Bacteriophage Nematicidal Activity

**Figure 2:**
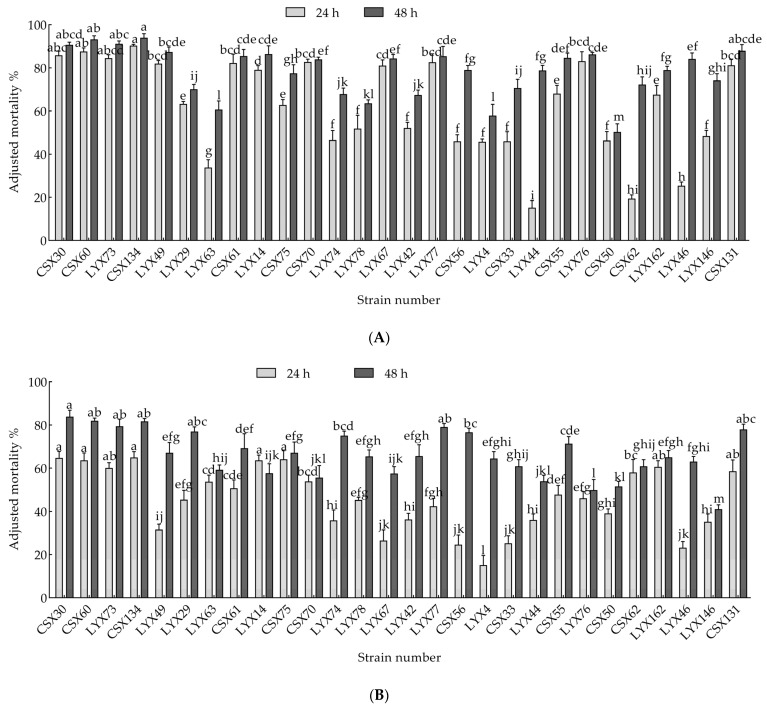
The nematicidal activity of bacterial strains is presented, including (**A**) 24-h and 48-h bacterial fermentation filtrates, and (**B**) 24-h and 48-h bacteriophage nematicidal activity. Significant variations in nematicidal efficacy among different strains were observed simultaneously (*p* < 0.05).

In the preliminary study, 48 strains exhibiting nematicidal activity were initially identified from the rhizosphere of horsetail grass. Among these, 28 strains demonstrated over 50% nematicidal activity in a 48-h screening. Subsequently, the nematicidal activity of bacterial fermentation filtrates and bacterial microorganisms was assessed, as depicted in Figure 2A,B. Overall, bacterial fermentation filtrates showed higher nematicidal activity compared to bacteriophages. Specifically, the fermentation filtrates of CSX60 and CSX134 strains (Figure 2A) exhibited significant nematicidal efficacy, achieving corrected mortality rates of 93.18% and 93.94% after 48 h, respectively. Meanwhile, bacteriophages CSX30, CSX60, and CSX134 (Figure 2B) displayed 48-h corrected mortalities exceeding 80%, with rates of 83.90%, 81.99%, and 81.87%, respectively. Consequently, CSX60 and CSX134 bacterial strains were selected for further experimentation.

#### 3.1.3. Molecular Biological Identification of Four Bacterial Strains

**Figure 3:**
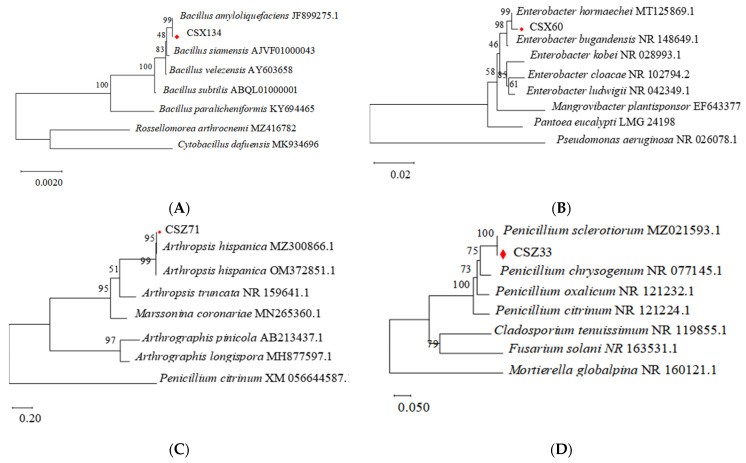
Phylogenetic tree of isolated strains: (**A**) strain CSX134 16S rDNA phylogenetic tree; (**B**) strain CSX60 16S rDNA phylogenetic tree; (**C**) strain CSZ71 ITS rDNA phylogenetic tree; (**D**) strain CSZ33 ITS rDNA phylogenetic tree.

From 3.1.1 and 3.1.2, strains CSX134, CSX60, CSZ71, and CSZ33 had the strongest nematicidal effect (93.94%, 93.18%, 91.54%, and 90.90%) and were selected for further studies. The bacteria CSX134 colonies on the LB medium were yellowish opaque colonies with a rough surface, elevated and irregular margins, positive Gram-stain, negative acetylmethyl methanol (V-P) test, and a negative indole test and methyl red (MR) test. Bacteria CSX60 formed rounded, creamy-white colonies on the LB solid medium, with a smooth, moist, and more transparent surface. The fungus CSZ71 inoculated into the center of the PDA medium was white and flocculent, and yellowish, with a texture ranging from powdery to granular; the fungus CSZ33 had white hyphae at the edges, which gradually became orange-yellow, and later turned dark green. The 16S-rDNA gene fragments of bacterial strains CSX134 and CSX60 were amplified, sequenced, and submitted to GenBank, with accession numbers PP389390 and PP389391, respectively. Subsequently, the 16S rDNA gene sequences of strains CSX134 and CSX60 were aligned, and a phylogenetic analysis was performed based on the 16S rDNA gene. The phylogenetic analysis revealed that strain CSX134 clustered closely with *Bacillus amyloliquefaciens*, forming a distinct sub-branch (Figure 3A) with a branch support value of 99.00%. Strain CSX60 showed a close phylogenetic relationship with *Enterobacter hormaechei*, sharing a common branch (Figure 3B) with a branch support value of 99.00%. Moreover, the ITS rDNA gene fragments of strains CSZ71 and CSZ33 were amplified, sequenced, and submitted to GenBank, with accession numbers PP389402 and PP389403, respectively. The gene sequences of strains CSZ71 and CSZ33 were aligned, and a phylogenetic analysis was performed based on the ITS rDNA gene. The phylogenetic analysis revealed that strain CSZ71 clustered closely with *Arthropsis hispanica*, forming a distinct sub-branch (Figure 3C) with a branch support value of 95.00%. Strain CSZ33 showed a close phylogenetic relationship with *Penicillium sclerotiorum*, sharing a common branch (Figure 3D) with a branch support value of 100.00%.

### 3.2. Determination of the Metabolites and Growth-Promoting Properties of Nematicidal Strains

The results are shown in Table 2. Strain CSX134 shows nitrogen-fixing ability, protease production, phosphorus solubilization ability, and IAA production capacity, as well as not producing chitin; strain CSX60 has nitrogen-fixing and potassicity solubilization abilities, and an IAA production capacity, and also does not produce chitin; strain CSZ71 has nitrogen-fixing ability, allows for cellulase production, and does not produce chitin, as well as possessing an IAA production capacity but no phosphorus solubilization ability; strain CSZ33 shows nitrogen fixation, protease, amylase, and phosphorus solubilization, and a potassium solubilization ability, and does not produce chitin. This means that all four strains of the bacterium have a certain pro-phytotic effect on the plants.

### 3.3. Screening of the Activity of Mixed Bacteria Against Pine Wood Nematodes

**Figure 4:**
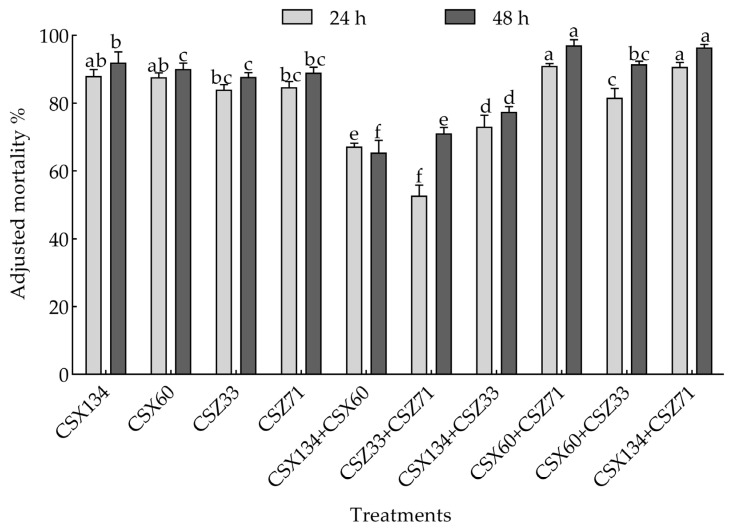
The nematicidal activity of mixed bacteria at 24-h and 48-h intervals is presented. Significant variations in nematicidal efficacy among the different strains were observed at each time point (*p* < 0.05).

After the screening process, strains CSX60, CSX134, CSZ71, and CSZ33 were chosen for a mixed bacterial strain. A 48-h nematocidal activity assay (Figure 4) showed that the fermentation filtrates of CSX134+CSZ71 and CSX60+CSZ71 had high nematode killing effects, with corrected mortality rates of 97.10% and 96.46%, respectively. Hence, these two groups of mixed bacteria were selected for further study.

### 3.4. Promotion of the Growth of P. massoniana Seedlings via Mixed Bacteria

**Figure 5:**
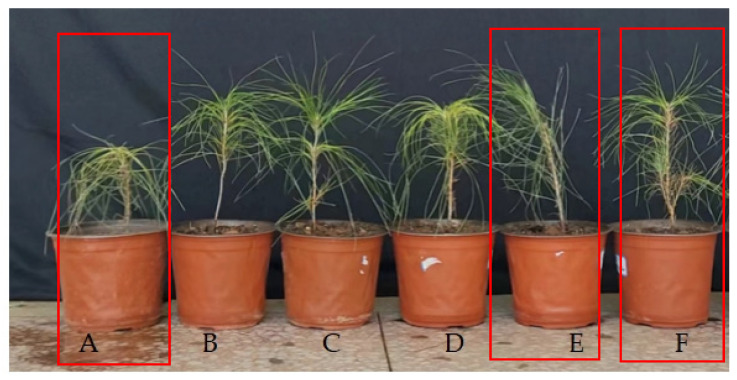
Growth of *P. massoniana* seedlings after 90 d under the following different treatments: (**A**) sterile water control; (**B**) single CSX134 strain treatment; (**C**) single CSX60 strain treatment; (**D**) single CSZ71 strain treatment; (**E)** double CSZ71+CSX134 composite treatment; (**F**) double CSZ71+CSX60 composite treatment.

*P. massoniana* seedling growth over 90 days of inoculation (Figure 5, Table 3) showed significant enhancement with CSX134, CSX60, CSZ71, CSZ71+CSX60, and CSZ71+CSX134 treatments. CSZ71 treatment increased plant height, above-ground fresh weight, above-ground dry weight, below-ground fresh weight, and below-ground dry weight by 10.70%, 38.55%, 29.71%, 22.22%, and 36.23%, respectively, compared to controls. CSX60 treatment increased these parameters by 23.05%, 76.99%, 65.22%, 123.66%, and 101.45%. CSX134 treatment showed increases of 21.51%, 34.05%, 3.99%, 48.75%, and 28.99%. CSZ71+CSX60 and CSZ71+CSX134 treatments significantly boosted growth by 34.16%, 109.61%, 77.54%, 144.80%, and 94.20% and by 23.46%, 104.27%, 59.78%, 130.47%, and 137.68%, respectively, compared to controls. These findings underscore the superior growth-promoting effects of combined CSZ71+CSX60 and CSZ71+CSX134 treatments over single CSZ71, CSX60, and CSX134 applications.

### 3.5. Influence of Fermentation Filtrate of Mixed Bacteria on the SOD Activity of P. massoniana

**Figure 6:**
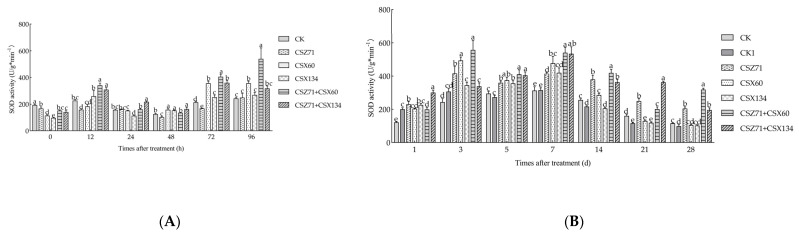
Variations in superoxide dismutase (SOD) activity within *P. massoniana* under different treatments. (**A**) Fermentation filtrate from various treatments during the initial four days; (**B**) fermentation filtrate from different treatments following nematode inoculation. CK (sterile water); CK1 (sterile water with pine wood nematodes). Error bars represent standard deviation. Different lowercase letters indicate significant differences between treatments (*p* < 0.05).

Superoxide dismutase (SOD) is a crucial antioxidant enzyme that is found widely in plants, enhancing resistance to aging, diseases, and environmental stressors. In *P. massoniana* (Figure 6A), the SOD activity after 12 h of inoculation surpassed that of the control in all groups except CSZ71. Control group SOD activity was lowest, peaking at 242.279 U/g·min^−1^ by 96 h. CSZ71 treatment steadily increased, reaching 246.652 U/g·min^−1^ at 96 h. Under CSX60 treatment, SOD initially decreased to 151.272 U/g·min·1 at 24 h, peaking at 355.288 U/g·min·1 at 72 h. CSX134 showed a low of 112.405 U/g·min·1 at 24 h, rising to 267.689 U/g·min·1 by 96 h. CSZ71+CSX60 hit a low of 135.156 U/g·min·1 at 48 h, peaking at 532.237 U/g·min·1 by 96 h. CSZ71+CSX134 saw a low of 162.730 U/g·min·1 at 48 h, peaking at 359.012 U/g·min·1 at 72 h, suggesting significant induction in vivo.

After 96 h of inoculation with pine nematodes, *P. massoniana* exhibited varying SOD enzyme activity. Figure 6B illustrates that, among the treatment groups, only CK1 (inoculated with pine nematodes only) showed lower SOD activity than CK. In the CK group, SOD activity initially increased, peaking at 312.45 U/g·min^−1^ on day 7 and then decreasing by day 28, except for the CSX134 and CSX60 treatment groups, where SOD activity remained higher than CK. In P. equisetifolia, SOD activity showed an increasing trend until day 5, and peaked at 415.45 U/g·min^−1^ on day 3 under CSZ71 treatment, at 493.67 U/g·min^−1^ on day 3 under CSX60 treatment, and at 419.03 U/g·min^−1^ on day 7 under CSX134 treatment. Overall, there was a significant induction of SOD activity in *P. massoniana* tissues treated with the mixed bacteria.

### 3.6. Influence of Fermentation Filtrate of Mixed Bacteria on PAL Activity of P. massoniana

**Figure 7:**
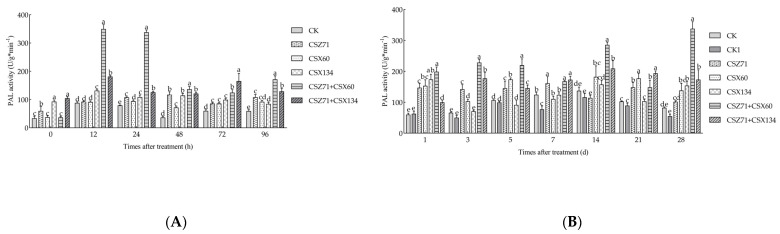
Variations in phenylalanine ammonia-lyase (PAL) activity within *P. massoniana* under different treatments. (**A**) Fermentation filtrate from various treatments during the initial four days; (**B**) fermentation filtrate from different treatments following nematode inoculation. CK (sterile water); CK1 (sterile water with pine wood nematodes). Error bars represent standard deviation. Different lowercase letters indicate significant differences between treatments (*p* < 0.05).

Phenylalanine ammonia-lyase (PAL) is a significant enzyme involved in the defense mechanisms of plants. In addition to its participation in phenylpropane metabolism, PAL contributes to the synthesis of compounds such as lignin and phytoalexins. An evaluation of PAL enzyme activity in *P. massoniana* (Figure 7A) revealed that all fermentation filtrate treatment groups exhibited higher enzyme activity compared to the control group within a 96 h period. Specifically, treatment with CSZ71+CSX60 and CSZ71+CSX134 composite fermentation filtrates resulted in higher PAL activity compared to the single-strain fermentation filtrate treatment group. Under CSZ71+CSX60 fermentation filtrate treatment, PAL activity displayed a biphasic trend, characterized by an initial rise, followed by a decline and a subsequent increase. The peak activity of 349.375 U/g·min^−1^ was observed at 12 h, while the lowest activity of 124.375 U/g·min^−1^ was recorded at 72 h, before experiencing a subsequent rise. In the case of CSZ71+CSX134 fermentation filtrate treatment, PAL activity exhibited two peaks at 12 h 181.667 U/g·min^−1^ and 72 h 165.625 U/g·min^−1^.

After 96 h period of fermentation filtrate treatment, subsequent inoculation with pine wood nematodes led to significant alterations in PAL enzyme activity (Figure 7B). The control group CK1, which was solely inoculated with pine wood nematodes, displayed an initial increase in PAL activity, followed by a decline, and maintained lower activity compared to the control group throughout the first 5 days. Under CSZ71+CSX60 fermentation filtrate treatment, PAL activity exhibited a similar biphasic pattern, with an initial rise, followed by a decline and a subsequent increase. Moreover, this treatment group consistently displayed higher activity compared to the control group and the CSZ71 and CSX60 single-strain treatment groups throughout the entire 28-day duration, with the peak activity of 337.744 U/g·min^−1^ being recorded at 28 days. Under the CSZ71+CSX134 fermentation filtrate treatment, PAL activity generally surpassed that of the control group and the nematode-inoculated control group. Notably, the PAL activity remained relatively stable under the CSZ71+CSX134 fermentation filtrate treatment and persisted until day 28. Conversely, the PAL activity under the CSX134 single-strain fermentation filtrate treatment reached its peak at 14 days 368.852 U/g·min^−1^, but displayed less stability, decreasing to its lowest point of 103.323 U/g·min^−1^ at 21 days. These results indicated that there was a significant induction of PAL activity in *P. massoniana* tissues under the treatment with the mixed bacteria.

### 3.7. Influence of Fermentation Filtrate of Mixed Bacteria on the CAT Activity of P. massoniana

**Figure 8:**
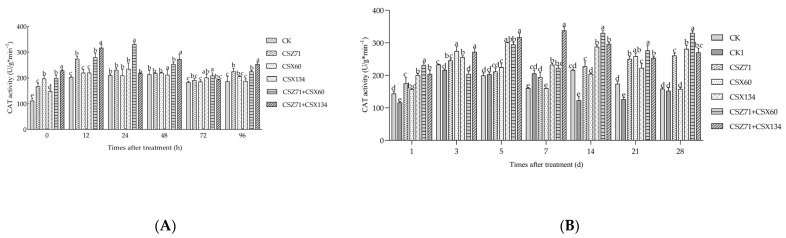
Variations in catalase (CAT) activity within *P. massoniana* under different treatments. (**A**) Fermentation filtrate from various treatments during the first four days; (**B**) fermentation filtrate from different treatments following nematode inoculation. CK (sterile water); CK1 (sterile water with pine wood nematodes). Error bars represent standard deviation. Different lowercase letters indicate significant differences between treatments (*p* < 0.05).

Catalase (CAT) plays a pivotal role in the respiration of organisms by providing an essential antioxidant defense mechanism and is primarily found in the chloroplasts and mitochondria of plants. Within *P. massoniana,* the activity levels of CAT in Figure 8A were examined under various treatment conditions. Following fermentation filtrate treatment, the enzyme activity in all treatment groups exhibited a notable increase compared to the control group over a 96 h period. Under the influence of CSZ71+CSX60 composite fermentation filtrate treatment, CAT activity displayed an initial rise followed by a decline, consistently surpassing the CK control group as well as the CSZ71 and CSX60 treatment groups. The maximum activity was recorded at 330.165 U/g·min^−1^ at 24 h, subsequently decreasing to 219.747 U/g·min^−1^ at 48 h, which was lower than that observed in the CSZ71 and CSX60 treatment groups. In the case of the CSZ71+CSX134 composite fermentation filtrate treatment, CAT activity was significantly higher than the CK control group but lower than the CSZ71 and CSX134 treatment groups. The CSZ71 treatment group reached its peak activity at 273.283 U/g·min^−1^ at 48 h, while the CSX134 treatment group exhibited its highest activity at 316.622 U/g·min^−1^ at 12 h. Subsequently, the CAT activity in the CSZ71+CSX134 composite fermentation filtrate treatment group outperformed the other groups at 72 h.

After 96 h of fermentation filtrate treatment, substantial variations in *P. massoniana* CAT enzyme activity were observed upon inoculation with pine wood nematodes in Figure 8B. Only the CK1 group, which was exclusively inoculated with pine wood nematodes, displayed an overall ascending and descending trend in CAT activity. In the CSZ71+CSX60 composite fermentation filtrate treatments, the CAT activity within *P. massoniana* surpassed that of the CK and CK1 control groups. Specifically, in the CSZ71+CSX60 composite fermentation filtrate treatment, CAT activity exhibited a fluctuating pattern, with enzyme activity exceeding that of the CK control group and the individual CSZ71 and CSX60 treatment groups over an extended duration. The highest activity of 330.537 U/g·min^−1^ was recorded at 14 days, while the lowest activity of 204.345 U/g·min^−1^ was observed at 3 days. Under the CSZ71+CSX134 fermentation filtrate treatment, CAT activity demonstrated an initial increase followed by a decrease and subsequent increase, consistently surpassing the CK control group, as well as the CSZ71 and CSX134 treatment groups, throughout the 28-day period. The enzyme activity peaked at 337.866 U/g·min^−1^ at 7 days, with significantly higher CAT activity being observed in the CSZ71 and CSX134 treatment groups compared to the CK and CK1 controls. These findings provide compelling evidence that there was a significant induction of CAT activity in *P. massoniana* tissues under the treatment with the mixed bacteria.

### 3.8. Effect of Fermentation Filtrate of Mixed Bacteria on the Control of Pine Wood Nematode Disease

**Figure 9:**
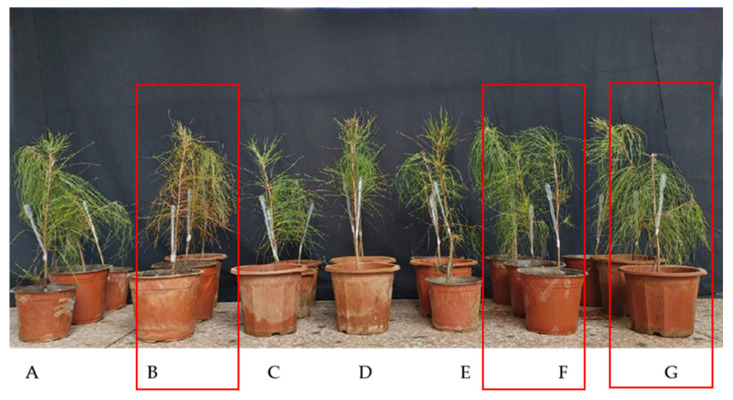
Growth of *P. massoniana* after 90 d under the following different treatments: (**A**) sterile water CK control; (**B**) CK1 only accepting pine wood nematode; (**C**) single CSX60 strain treatment; (**D**) single CSX134 strain treatment; (**E**) single CSZ71 strain treatment; (**F**) double CSX134+CSZ71 composite treatment; (**G**) double CSX60+CSZ71 composite treatment.

By day 28 of pine wood nematode inoculation, Figure 9 demonstrates that the mixed bacterial fermentation filtrate CSZ71 + CSX60 and CSZ71 + CSX134 exhibited superior control efficacy. Pine needles of *P. massoniana* treated with these filtrates remained green, whereas those only inoculated with pine wood nematode displayed yellowing and reddening symptoms. In accordance with the methodology proposed by Xue et al. [41], the disease severity classification and disease index calculation were performed for treatment combinations using a double application of CSX134 and CSZ71 and double application of CSX60 and CSZ71. The disease index was computed using the following formula: disease index = ∑ (proportion of trees in each disease severity class × representative value of the severity). As shown in Table 4, all the treatment combinations showed significant results regarding disease control. Under the 28-day treatment conditions, the CSZ71+CSX60 combination demonstrated the highest efficacy rate of 75.07%, followed by CSZ71+CSX134, with an efficacy rate of 69.65%. The efficacy rates for CSX134, CSZ71, and CSX60 were 60.06%, 54.53%, and 52.15%, respectively. Therefore, the treatment combinations ranked in descending order of efficacy are as follows: CSZ71+CSX60 > CSZ71+CSX134 > CSX60 > CSX134 > CSZ71.

### 3.9. Effects of Fermentation Filtrate of Mixed Bacteria on the Expression Levels of Related Genes of P. massoniana

**Figure 10:**
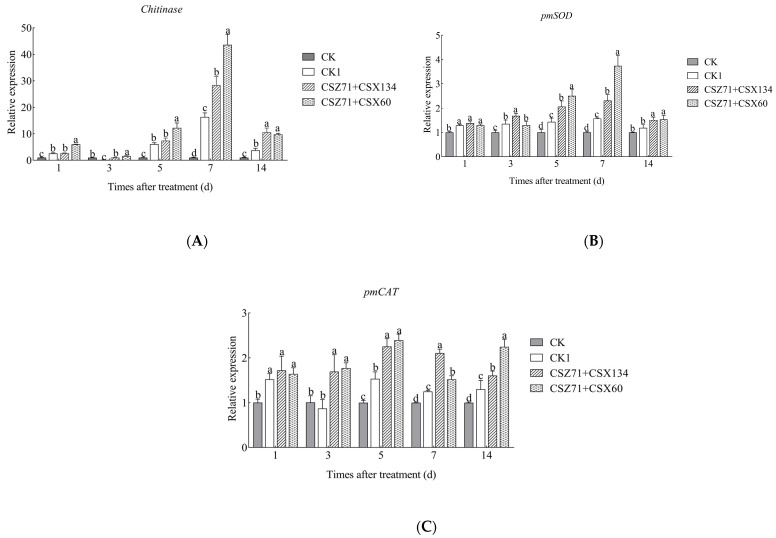
Expression of resistance genes in needles of *P. massoniana* under the following different treatments: (**A**) chitinase; (**B**) polyphenol oxidase (SOD); (**C**) catalase (CAT). CK: *P. massoniana* treated with sterile distilled water; CK1: *P. massoniana* treated with pine wood nematode inoculation. Error bars represent standard deviation. Different lowercase letters indicate significant differences between treatments (*p* < 0.05).

Based on the results of the defense enzyme activity assays and the efficacy chart, we carefully selected the CSX60+CSZ71 and CSX134+CSZ71 combination treatments to investigate their influence on the expression levels of defense-related genes in *P. massoniana*. In this study, we aimed to assess the expression profiles of three genes, namely Chitinase, PmSOD, and PmCAT, which are known to be associated with disease progression, defense response enzyme synthesis, and stress tolerance in *P. massoniana*. Specifically, we examined the expression patterns following treatment with the composite fungal strains CSZ71+CSX60 and CSZ71+CSX134. As illustrated in Figure 10A, it is noteworthy that the expression level of the Chitinase gene displayed a biphasic trend over the entire experiment. Notably, the CSZ71+CSX60 treatment group exhibited a peak in Chitinase expression at 7 days, demonstrating a 43.5 times increase compared to the control group CK and a 2.68 times increase compared to the CK1 control group. Subsequently, the expression level declined after 14 days. Overall, the CSZ71+CSX60 treatment group exhibited significantly higher chitinase expression levels compared to the CK and CK1 control groups. Similarly, the CSZ71+CSX134 treatment group also exhibited a peak Chitinase expression at 7 days, with expression levels 28.3 times higher than the CK group and 1.73 times higher than the CK1 control group.

Based on the findings depicted in Figure 10B, it is evident that the expression level of the pmSOD in *P. massoniana* at 14 d under the treatment with CSZ71+CSX60 showed a trend of first rising and then declining later, and reached its peak at 7 d, which was 2.4 times later than control group CK, and 1.6 times later than control group CK1. The expression of the gene after 7 d was the lowest, but was still higher than the expression of the treatment groups CK and CK1. The overall gene expression of pmSOD in the CSZ71+CSX134 treatment group showed a trend of increasing and then decreasing, with a peak at 5 d, when it was 2.3 times that of the control CK and 1.53 times that of CK1, respectively.

From the data shown in Figure 10C, pmCAT gene expression in ponytail pine treated with CSZ71+CSX60 can be observed to initially increase, peaking on day 5 at 2.1 times higher than the CK group and 1.6 times higher than CK1, then decreasing thereafter. Notably, at 7 days, the expression level was the lowest among the observed time points but remained higher than that of the CK and CK1 control groups. In the CSZ71+CSX134 treatment group, the overall expression pattern of the pmCAT gene exhibited an initial increase followed by a subsequent decrease. The highest expression level was recorded at 5 days, exhibiting a 2.3 times increase compared to the CK group and a 1.5 times increase compared to the CK1 control group.

## 4. Discussion

In recent years, there has been an increasing focus on finding sustainable and environmentally friendly methods for controlling pine wilt disease, with biological control emerging as a promising approach. Microbes exert their control by directly or indirectly inhibiting and eradicating pathogenic bacteria through various biocontrol mechanisms. Additionally, microorganisms produce a wide array of hydrolytic enzymes, such as proteases, chitinases, and cellulases, which facilitate the degradation of pathogen tissues [42,43,44,45,46,47]. In addition, the induction of plant defense response and promotion of host plant growth is also one of the biocontrols. *Bacillus licheniformis* YZCUO202005, isolated by Medison, inhibited the growth of five pathogenic fungi; they have the ability to secrete IAA, proteases, amylases, and cellulases, and can promote host growth and disease resistance under greenhouse conditions [48]. In this study, CSX134, CSX60, CSX71, and CSZ33 were shown to have different PGP capacities, while an in vitro test showed that different strains of bacteria, when used in the root irrigation treatment of *P. massoniana* plant height, significantly increase the aboveground dry weight, aboveground fresh weight, underground dry weight, and underground fresh weight of soil urease, as well as soil acid phosphatase. The CSX71+CSX60, CSX134+CSZ71 mixed bacterial fermentation filtrate treatments of *P. massoniana* appeared to have the strongest effects. Meena et al. found that co-inoculation of *Pseudomonas striata* and the endophytic fungus *Piriformospora indica* had a synergistic effect on population build-up and plant dry mass of *Cicer arietinum* as compared to inoculation alone [49]. Several studies have reported the pivotal role played by specific bacterial genera, including *Bacillus*, *Serratia*, *Enterobacter* sp., *Aspergillus*, and *Estrella*, which demonstrate nematocidal activity through parasitic mechanisms or the production of toxic compounds [46,47,50]. Notably, *Enterobacter* sp. strain AA4 has exhibited remarkable nematocidal activity against pine wood nematode and demonstrated significant biocontrol efficacy against *Bursaphelenchus xylophilus*-induced pine wilt disease [51]. Furthermore, researchers have successfully isolated strain LCB-3 from plants, which exhibits potent nematocidal activity and yielded bioactive compounds with nematocidal properties from its fermentation broth [52].

Bailey et al. conducted a study that revealed the ability of endophytic fungi from the *Trichoderma* genus to induce plant resistance through the production of xyloglucans or other elicitors [53]. Furthermore, researchers have observed that Indian pear-shaped spores can enhance the resistance of host plants. Mendoza and Sikora [54] demonstrated that the combined application of non-toxic *Fusarium oxysporum*, which induces plant resistance, and robust spore-forming *Bacillus firmus*, which exhibits strong larvicidal effects on root-knot nematode second-stage juveniles, effectively reduces the population density of banana root-lesion nematodes. Additionally, Peng et al. significantly decreased the incidence of root-knot nematode disease by utilizing a fermentation broth derived from a blend of two *Streptomyces* strains and three endophytic *bacterial* strains [55]. In our experimental investigations, we isolated and screened four strains of bacteria, CSX134 (*Bacillus myloliquefaciens*) and CSX60 (*Enterobacter hormaechei*), fungi CSZ71 (*Arthropsis hispanica*), and CSZ33 (*Penicillium sclerotiorum*), which showed significant nematicidal activity. Subsequently, we conducted a composite screening assay using fermentation filtrates, whereby the nematocidal activity of the CSZ71+CSX60 and CSZ71+CSX134 combinations surpassed that of the individual strains. Furthermore, a pot experiment employing the fermentation filtrates of CSZ71+CSX60 and CSZ71+CSX134 confirmed their superior efficacy in the biological control of pine wood nematode disease in *P. massoniana*.

When plants face pathogen attacks, they activate defense responses. Systemic acquired resistance (SAR) and induced systemic resistance (ISR) are key mechanisms in plants’ defense against pathogens. SAR relies on the salicylic acid (SA) pathway, while ISR is primarily mediated by the jasmonic acid (JA) and ethylene (ET) pathways [56]. Wu et al. conducted a study demonstrating that the treatment of two-year-old *P. massoniana* with a suspension or inactivated suspension of *Bacillus cereus* NJSZ-13 led to an increase in the activity of defense enzymes, including peroxidase (POD), superoxide dismutase (SOD), and phenylalanine ammonia-lyase (PAL), indicating the strain’s potential to induce resistance against pine wood nematode disease [57]. Chen et al. observed the involvement of PAL in modulating the stress response during pine wood nematode infestation in *P. massoniana*. Furthermore [58], Wang et al. revealed a significant enhancement in the activity of antioxidant enzymes and PAL following the invasion of Japanese larch by pine wood nematodes [59]. PAL assumes a prominent role as an essential defense enzyme in plants, closely linked to systemic resistance. SOD and CAT participate in the safeguarding of plants against potential hazards posed by reactive oxygen species (ROS) and are intimately entwined with the host plant’s defense against pathogenic incursions. The experiments demonstrated that fermentation filtrates from various treatments induced plant disease resistance in *P. massoniana*. Treatment with composite fermentation filtrates of CSZ71+CSX60 and CSZ71+CSX134 significantly increased PAL, POD, and CAT enzyme activities over 28 days. Plant disease resistance genes detect nematode-secreted effectors, triggering signaling pathways and various defense responses. The initial defense response involves a burst of reactive oxygen species (ROS). Normally, plants maintain a dynamic equilibrium of ROS production and removal, but stimulation from a foreign organism can disrupt this balance, causing plant damage [60]. Xie observed a ‘first increase and then decrease’ trend in the expression of ROS-related genes PmSOD and PmCAT in drug-resistant and sensitive *P. massoniana* inoculated with pine wood nematode [38]. Zheng et al. explored the transcriptomic response of disease-resistant *P. massoniana* GD5 seed sources to pine wood nematode, revealing the significant upregulation of six chitinase genes involved in the aldehyde dehydrogenase pathway of sennosine and nucleotide sugar metabcompoundolism [61]. In the present experiment, the expression levels of chitinase, pmSOD, and pmCAT in the fermentation filtrate of CSZ71+CSX60 and CSZ71+CSX134 bacteria in *P. massoniana* showed a tendency to increase and then decrease over 14 days, and their expression was higher than that in the control group. Previous studies have shown that resistance was cultivar-specific and that the increased activity of defense-related enzymes depended on factors such as the inducing agent, plant genotype, and challenging pathogen [62]. Therefore, the expression levels of defense-related genes are crucial in Masson pine resistance to pine wood nematode, consistent with our observations, but their relevance and mechanism require further validation.

The topics proposed for further research include the following: (1) the components of secondary metabolites of the mixed bacteria can be analyzed to identify substances with resistance-inducing and growth-promoting abilities; (2) changes in gene expression in the fermentation filtrate of the mixed bacteria in pinus sylvestris can be comprehensively analyzed through sequencing the transcriptome to identify differentially expressed genes and associated signaling pathways, as well as the initiated signal transduction pathways.

## 5. Conclusions

In this study, strains CSX134, CSX60, CSZ71, and CSZ33, which were shown to possess a high killing rate of pine wood nematodes, were screened and functionally tested from the inter-root soil in *P. massoniana* to synthesize a variety of metabolic substances and secrete a series of pro-biotic substances. Strain CSX134 was identified as *Bacillus amyloliquefaciens*, strain CSX60 was identified as *Enterobacter hormaechei*, strain CSX71 was identified as *Arthropsis hispanica*, and strain CSZ33 was identified as *Penicillium sclerotiorum*. The results of the screening of the mixed bacterial-killing line showed that CSX134+CSZ71 and CSX60+CSZ71 had significantly higher nematicidal activity than the single-bacterial nematicidal activity. In vitro experiments demonstrated that pre- and post-inoculation with pine nematode, the PAL, SOD, and CAT activities of the CSX134+CSZ71 and CSX60+CSZ71 mixed-bacteria fermentation filtrates were significantly higher than the controls in *P. massoniana*-treated pines. Furthermore, these filtrates significantly induced the expression of defense-related enzymes Chitinase, pmPOD, and pmCAT. These study results indicate that the fermentation filtrates of the mixed cultures CSX134+CSZ71 and CSX60+CSZ71 may control pine wilt disease in *P. massoniana* by enhancing the activity of defense-related enzymes and the expression levels of associated genes.

## Figures and Tables

**Table 1:** Details of test treatment.

Treatment	Processing Details/Pot
Treatment CK	CK 100 mL sterile water; root irrigation every 30 days.
Treatment 3: CSX134	CSX134 bacteria were added to 100 mL of fermentation filtrate, and roots were irrigated every 30 days.
Treatment 4: CSX60	CSX60 bacteria were added to 100 mL of fermentation filtrate, and roots were irrigated every 30 days.
Treatment 5: CSZ71	Add 100 mL of fermentation filtrate of CSZ71 fungus, and irrigate the roots every 30 days.
Treatment 6: CSZ71+CSX134	Add 100 mL of CSZ71+CSX134 mixed bacteria in a 1:1 mixture with fermentation filtrate, rooting every 30 days.
Treatment 7: CSZ71+CSX60	Add 100 mL of CSZ71+CSX60 mixed bacteria in a 1:1 mixture with fermentation filtrate, rooting every 30 days.

**Table 2:** Plant growth-promoting (PGP) substances and extracellular enzymes of four strains.

Item	Substances	CSX134	CSX60	CSZ71	CSZ33
PGP traits	IAA	+	+	+	−
	Hyperkalosis	−	+	−	+
	Organic phosphate	+	+	+	+
	Nitrogen fixation	+	+	+	+
Extracellular enzymes	Cellulase	+	−	−	+
	Chitinase	−	−	−	−
	Protease	+	+	+	+
	Amylase	+	−	−	+

**Table 3:** Effect of different treatments on the growth of *P. massoniana* seedlings.

Treatments	Shoot Fresh Weight (gt)	Shoot Dry Weight (g)	Root Resh Weight (g)	Root Dry Weight (g)
CK	2.81 ± 0.15 d	0.92 ± 0.10 c	0.93 ± 0.06 c	0.23 ± 0.01 b
CSZ71	3.89 ± 0.27 c	1.19 ± 0.05 b	1.14 ± 0.09 ab	0.31 ± 0.05 b
CSX60	4.97 ± 01.7 b	1.52 ± 0.03 a	2.08 ± 0.16 a	0.46 ± 0.03 a
CSX134	3.77 ± 0.10 c	0.96 ± 0.04 bc	1.38 ± 0.10 b	0.30 ± 0.01 b
CSZ71+CSX60	5.89 ± 0.06 a	1.63 ± 0.06 a	2.28 ± 0.03 a	0.45 ± 0.03 a
CSZ71+CSX134	5.74 ± 0.25 a	1.47 ± 0.13 a	2.14 ± 0.10 a	0.55 ± 0.02 a

Different lowercase letters in the same column indicate significant differences between treatments (*p* < 0.05).

**Table 4:** Biological control of pine nematode disease in *P. massoniana* using a nematicide complex.

Treatments	Disease Index (%)	Biocontrol Efficacy (%)
CK	0	–
CK1	77.50 ± 3.82 a	–
CSX60	30.83 ± 1.39 bc	60.06 ± 2.80 bc
CSX134	35.00 ± 2.89 b	54.53 ± 5.36 d
CSZ71	36.67 ± 3.00 b	52.77 ± 2.37 d
CSX134+CSZ71	23.33 ± 2.20 cd	69.65 ± 4.00 ab
CSX60+CSZ71	19.17 ± 1.67 d	75.07 ± 3.07 a

Different lowercase letters in the same column indicate significant differences between treatments (*p* < 0.05).

## Data Availability

The original contributions presented in the study are included in the article; further inquiries can be directed to the corresponding authors.

## Abbreviations

Superoxide dismutase, SOD; Catalase, CAT; Phenylalanine ammonialyase, PAL; Proso millet peroxidase gene, pmPOD; Catalasegene, pmCAT.
